# Efficacy of pulpotomy for permanent teeth with carious pulp exposure: A systematic review and meta-analysis of randomized controlled trials

**DOI:** 10.1371/journal.pone.0305218

**Published:** 2024-07-05

**Authors:** Wenjun Li, Bo Yang, Jing Shi

**Affiliations:** Department of Oral Medicine, Shanxi Provincial People’s Hospital, Taiyuan, Shanxi Province, China; Universidade de Trás-os-Montes e Alto Douro: Universidade de Tras-os-Montes e Alto Douro, PORTUGAL

## Abstract

This meta-analysis aims to assess the success rate of pulpotomy in the treatment of permanent teeth with carious pulp exposure and to compare the efficacy of different capping materials. Randomized controlled trials were searched in PubMed, EMBASE, Web of Science, Clinicaltrial.gov, and Cochrane Library until August 31, 2023. The pooled success rate was estimated in the overall population and in subgroups. Additional analyses comparing different capping materials using odds ratio (OR) and 95% confidence interval (95%CI) were performed. The certainty of evidence was graded using the GRADE approach. A total of 25 randomized trials with an average follow-up duration ≥ 12 months were finally included. The overall success rate of pulpotomy was 86.7% (95%CI: 82.0–90.7%). The success rate was not significantly affected by root development, pulpotomy type, and follow-up duration. Teeth with irreversible pulpitis had a relatively lower success rate than teeth with normal pulp or reversible pulpitis (82.4% [95%CI: 74.6–89.0%] *vs* 92.0% [95%CI: 87.9–95.4%], P = 0.013). Directly compared to conventional calcium hydroxide, mineral trioxide aggregate (88.2% *vs* 79.1%, OR = 2.41, 95%CI: 1.28–4.51, P = 0.006) and Biodentine (97.5% *vs* 82.9%, OR = 6.03, 95%CI: 0.97–37.6, P = 0.054) had higher successful rates. No significant difference between MTA and other biomaterials was found. The results were graded as very low to low certainty of evidence. In conclusion, pulpotomy is an effective treatment of permanent teeth with carious pulp exposure. Mineral trioxide aggregate and Biodentine can be recommended with more favorable outcomes as capping materials.

## Introduction

Root canal treatment (RCT), completely removing the coronal and radicular pulp tissue, is the traditional treatment choice for cariously exposed teeth, particularly in mature permanent teeth [1]. Yet, histological studies suggested that bacterial infection may be limited to the coronary pulp tissue without involving the radicular pulp tissue, questioning the necessity of complete removal of pulp tissues [2]. Moreover, despite a high success rate, RCT compromises the structural and functional integrity of dentin-pulp complex.

Vital pulp therapy (VPT) aims to preserve the pulp vitality and the healthy pulp tissues, stimulate the regeneration of dentin-pulp complex, and maintain its function like proprioception, mechanoreceptor function, innervation and vascularization [3, 4]. The preservation of pulp vitality is important for the treatment of permanent teeth with carious pulp exposure. Functional experiments demonstrate that, in vital pulp tissues, odontoblasts, fibroblasts, and immune cells together with functionally related molecules jointly participate in the immune defense, maintain the reparative capacity, and promote the healing of dental pulp tissue [5].

VPT encompasses indirect or direct pulp capping (DPC), partial pulpotomy (PP), and full pulpotomy (FP). Pulpotomy preserves pulp vitality by partially or completely removing the inflamed coronal pulp tissue followed by the placement of a bioactive pulp dressing material after sufficient hemostasis [6]. With comparable success rate, pulpotomy has several advantages over RCT as it is more cost-effective, easier to perform technically, less time-consuming, and minimally invasive [7, 8]. In permanent teeth with deep varies and pulp exposure, pulpotomy, either partial pulpotomy or full pulpotomy, demonstrates higher success rates compared with direct pulp capping [9].

Traditionally, the indication of pulpotomy is limited to carious primary teeth or traumatized permanent teeth [10, 11]. With the better understanding of pulp repair process and the development of more biocompatible, anti-inflammatory and osteo-inductive capping materials, pulpotomy is now more and more frequently adopted for treatment of permanent teeth with cariously exposed pulp in various clinical scenarios, such as mature teeth with irreversible pulpitis, with different pulpotomy procedures and capping materials [12–15]. Yet, the success rates widely ranged from 77% to 100% [12–15]. Whether the selection of patients according to root development and preoperative pulp diagnosis and the choice of pulpotomy procedures and pulp dressing materials may influence the efficacy of pulpotomy remain inconclusive. Several previous meta-analyses focused on the overall success rate and explored the influential factors, but they had a small sample size, comprised traumatic teeth, or included observational studies [16–18]. The others only focused on the comparison of different capping materials [19–21]. In recent years, increasing evidence is accumulated by more and more randomized controlled trials [22–24]. Here, we perform a systematic review and meta-analysis of randomized controlled trials to comprehensively assess the efficacy of pulpotomy in permanent teeth with carious pulp exposure in overall and in different clinical scenarios by root maturation, pulp diagnosis, capping materials, and pulpotomy procedures.

## Materials and methods

### Literature search and selection criteria

This systematic review and meta-analysis was conducted in compliance with Preferred Reporting Items for Systematic review and Meta-analysis guideline (S1 Checklist) [25]. Comprehensive literature search was performed in electronic databases, including PubMed, EMBASE, Scopus, Web of Science, Clinicaltrial.gov, and Cochrane Library, prior to August 31, 2023 using the following search terms: (vital pulp OR vital pulp therapy OR pulpotomy) AND (permanent OR mature OR immature) AND (randomized OR randomised). A grey literature search was also performed through Google Scholar. No language restriction was applied. The reference lists of relevant reviews, meta-analysis, and included trials were manually searched for more eligible studies that were potentially missed by literature search.

The inclusion criteria were summarized according to the PICOS framework as follows: P (participant): permanent teeth with carious pulp exposure treated with pulpotomy; I (intervention) and C (comparison): comparing at least two different capping materials, or comparing FP *vs*. PP; O (outcome): overall success at follow-up of ≥ 12 months; S (study design): randomized controlled trial. Here, the overall success was a composite outcome defined as achievement of both clinical success and radiographic success. Clinical success was defined as the absence of clinical manifestations including pain and sensitivity to percussion and palpation. Radiographic success was defined as the absence of periapical radiolucency.

Studies were discarded according to the following exclusion criteria: (1) including primary teeth; (2) receiving other vital pulp therapy such as indirect pulp capping and direct pulp capping; (3) including traumatized teeth; (4) only reporting one of clinical success and radiographic success, or reporting insufficient data to calculate the overall success rate; (5) case series, case reports, prospective and retrospective non-randomized studies, reviews, meta-analysis, animal studies, and experimental studies.

### Data extraction

We extracted the following details of included randomized trials: first author, publication year, type of pulpotomy, sample size, age range, type of teeth, root maturation, preoperative pulpal diagnosis, capping materials, hemostasis solution, type of final restoration, duration of follow-up, clinical success rate, radiographic success rate, and overall success rate.

### Methodology assessment

Methodology assessment was performed by using the Cochrane Collaboration’s tool for assessing risk of bias [26]. Selection bias (random sequence generation, allocation concealment), performance bias (blinding of participants and personnel), detection bias (blinding of outcome assessment), attrition bias (incomplete outcome), reporting bias (selective reporting), and other bias were judged at low, unclear, and high risk. Two independent authors performed literature search and selection, data extraction, and risk of bias assessment. Conflicts, if occurred, were resolved by further discussion.

### Statistical analysis

The present meta-analysis was performed using STATA 16.0 (StataCorp, TX, US). We used *I*^*2*^ statistic to assess between-study heterogeneity with *I*^*2*^ < 50% and ≥ 50% indicating low and high heterogeneity, respectively. A fixed-effect model was applied for meta-analysis with low heterogeneity, and a random-effect model was used for meta-analysis with high heterogeneity. The efficacy of pulpotomy in permanent teeth with carious pulp exposure was estimated by calculating the proportion of overall success with corresponding 95% confidence interval (95%CI) using the *metaprop* package in STATA [27]. Direct comparison of the efficacy of different capping agents was also performed. Pooled odds ratio (OR) with 95%CI was calculated in comparisons with at least two available trials. Subgroup analyses stratified by root maturation (mature, immature, mixed), preoperative pulpal diagnosis (irreversible pulpitis, normal pulp or reversible pulpitis, unspecified or mixed diagnosis), type of pulpotomy (FP, PP), hemostasis time (6 min or less, 10 min or more, unknown), and follow-up duration (12 months, 24 months) were conducted. Further subgroup analyses were done according to the combinations of root maturation, pulpal diagnosis, and type of pulpotomy. Meta-analysis was performed for the association of success rate with mean age and sample size. Publication bias was assessed in for meta-analysis comparing the efficacy of different capping materials by viewing the symmetry of funnel plot and Egger’s test. P value < 0.05 indicated statistical significant.

### Certainty of evidence

The certainty of evidence was assessed by using the Grading of Recommendations Assessment, Development and Evaluation (GRADE) approach [28]. The certainty was assessed based on several domains, including risk of bias, inconsistency, indirectness, imprecision, and publication bias. The overall certainty of evidence was graded as very low, low, moderate, and high. This assessment was performed by two independent authors. If there was a discrepancy, a consensus was reached by further discussion.

## Results

### Description of characteristics of included trials

**Fig 1:**
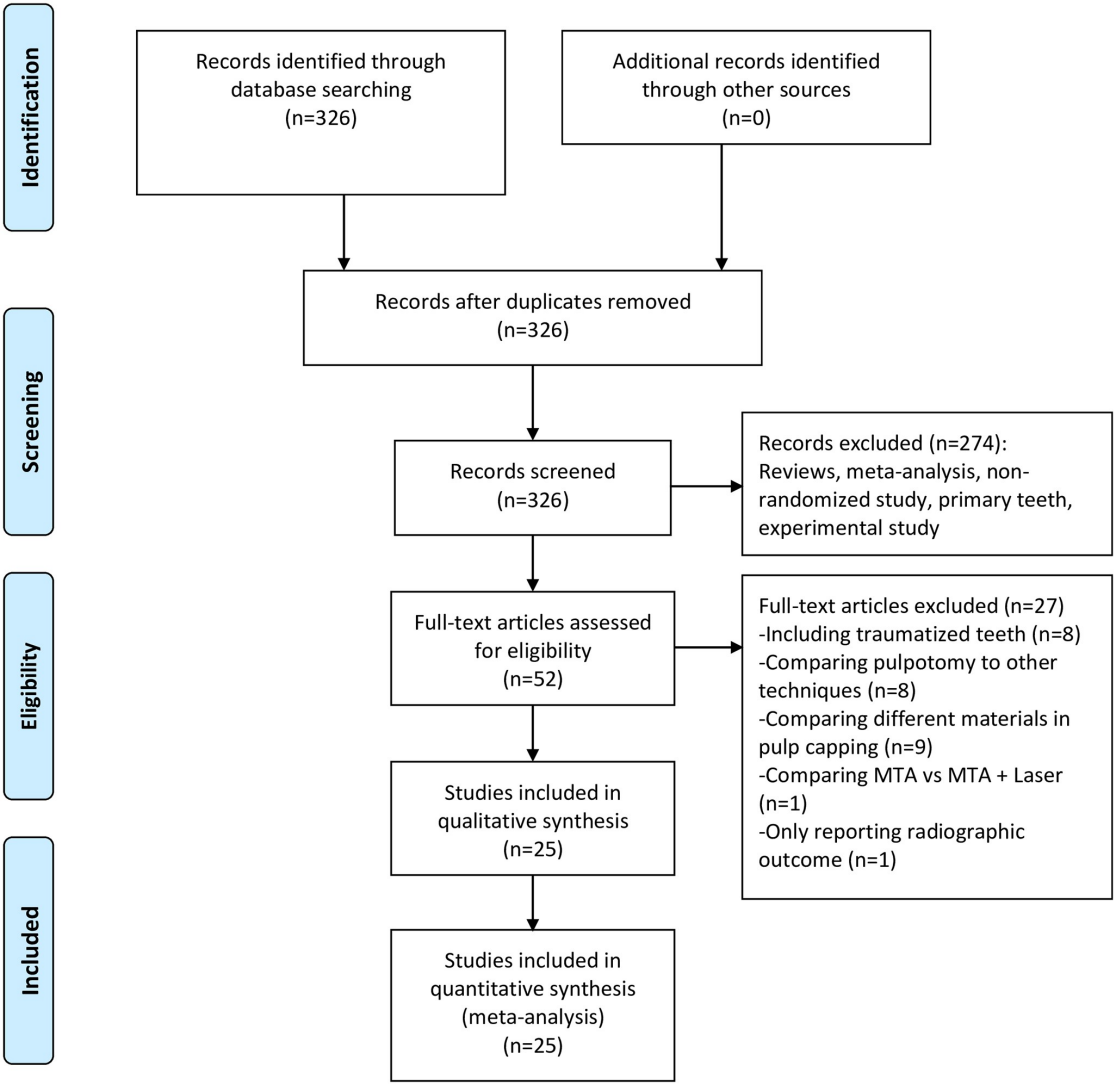
Flowchart of literature search and selection.

Comprehensive literature search identified 326 unique articles with 52 articles remained for further eligibility assessment. Through reviewing the full text, 27 articles were discarded due to various reasons listed in S1 Table. Finally, a total of 25 RCTs were eligible for qualitative and quantitative synthesis (Fig 1) [22–24, 29–50]. The sample size ranged from 24 to 413, and the total sample size was 2085 teeth. All RCTs were in a parallel design except for 1 in a split-mouth design [40]. Three trials compared PP to FP [22, 45, 46], and the others compared the efficacy of different capping materials in pulpotomy. Most of the trials assigned participants to 2 arms of capping materials, 5 assigned to 3 arms [31, 33, 44, 49, 50], and 1 assigned to 4 arms [23]. According to root maturation, 12 trials included only mature permanent teeth, 10 included only immature teeth, and 3 included both [36, 38, 47]. A preoperative irreversible pulpitis diagnosis was necessary for the recruitment of participants in 14 trials. Whereas, the inclusion criteria for preoperative pulpal diagnosis in the other trials varied, including normal pulp, reversible pulpitis, unspecified or mixed diagnosis. In 10 trials, only teeth achieving hemostasis within 6 min or less following pulpotomy were further capped with biomaterials. The hemostasis time cutoffs were 10 min or more in 9 studies and unknown in the rest. Two studies reported the success rate at the last follow-up (mean follow-up > 24 months) [36, 38]. The other trials reported postoperative outcomes at 12 (n = 14), 18 (n = 2), and 24 (n = 7) months of follow-ups. The baseline characteristics of all included RCTs were summarized in Table 1.

**Table 1:** Baseline characteristics of trials included in meta-analysis.

Study	Pulpotomy	Root maturation	Pulpal diagnosis	Capping materials (n)	Hemostasis time, min	Age range (mean ± SD, years	Follow-up, months	Success rate, %
Qudeimat, 2007	PP	Mixed	Normal pulp/reversible pulpitis	MTA (32), CH (32)	NA	6.8–13.3 (10.3 ± 1.8)	34.8 ± 4.4	MTA: 92.3%; CH: 91.3%
Nosrat, 2012	FP	Immature	Unspecified	MTA (25), CEM (26)	5–10	6–10 (8.38 ± 1.06)	12	MTA: 81.5%; CEM: 78.9%
Asgary, 2013	FP	Mature	Irreversible pulpitis	MTA (208), CEM (205)	NA	9–65 (26.4)	12	MTA: 87.2%; CEM: 92.2%
Chailertvanitkul, 2014	PP	Immature	Reversible pulpitis	MTA (44), CH (40)	1–2	7–10	24	MTA: 93.2%; CH: 87.5%
Keswani, 2014	FP	Immature	Unspecified	MTA (31), PRF (31)	2–3	6–12 (7.8)	24	MTA: 80.8%; PRF: 88.9%
Kumar, 2016	FP	Mature	Irreversible pulpitis	MTA (19), CH (18), PRF (17)	NA	14–32 (21.5)	12	MTA: 44.4%; CH: 37.5%; PRF: 35.7%
Taha, 2017	PP	Mature	Irreversible pulpitis	MTA (27), CH (23)	2–6	20–52 (30.3 ± 9.6)	24	MTA: 84.6%; CH: 43.5%
Ozgur, 2017	PP	Immature	Unspecified	MTA (40), CH (40)	5	6–13 (8.6)	24	MTA: 97.3%; CH: 97.4%
Eppa, 2018	FP	Immature	Unspecified	MTA (20), Triple Antibiotic Paste (20), Abscess Remedy (20)	5	6–14	24	MTA: 100%; Triple Antibiotic Paste: 100%; Abscess Remedy: 80.0%
Uesrichai, 2019	PP	Mixed	Irreversible pulpitis	MTA (37), Biodentine (32)	10	6.4–16.9 (10 ± 2.1)	32.2 ± 17.9	MTA: 91.9%; Biodentine: 86.7%
Alawwad, 2020	FP	Immature	Irreversible pulpitis	MTA (12), PRF (12)	10–15	6–8 (6.9)	12	MTA: 50.0%; PRF: 41.7%
Abuelniel, 2021	FP	Immature	Unspecified	MTA (30), Biodentine (30)	5	7–8 (7.3 ± 1.1)	18	MTA: 80.0%; Biodentine: 80.0%
Ahmed, 2021	FP	Immature	Unspecified	MTA (25), Potassium nitrate in polycarboxylate cement (25)	5–10	6–9 (7.7)	12	MTA: 92.0%; Potassium nitrate in polycarboxylate cement: 92.0%
Uyar, 2021	PP	Immature	Normal pulp/reversible pulpitis	MTA (18), Biodentine (18), CH (18)	2	6–13 (7.9)	12	MTA: 94.4%; CH: 72.2%; Biodentine: 94.4%
Asgary, 2022	FP	Mature	With or without irreversible pulpitis	MTA (55), CEM (51)	5	14–60 (31.7)	24	MTA: 100%; CEM: 97.9%
Taha, 2022	FP	Mature	Reversible and irreversible pulpitis	MTA (50), Biodentine (50), TotalFill BC(64)	6	10–70	12	MTA: 91.8%; Biodentine: 93.3%; TotalFill BC: 91.9%
Ramani, 2022	PP, FP	Mature	Irreversible pulpitis	MTA (106)	NA	18–40 (23.32 ± 4.85)	12	MTA: 85.1%
Doranala, 2021	FP	Mature	Irreversible pulpitis	CH (20), EndoSequence (20), PRF (20)	NA	15–55 (24.4)	12	CH: 65.0%; PRF: 85.0%; EndoSequence: 75.0%
Baranwal, 2022	PP, FP	Mature	Irreversible pulpitis	Biodentine (66)	10	18–40	12	Biodentine: 87.0%
Jassal, 2023	PP, FP	Mature	Irreversible pulpitis	Biodentine (50)	10	≥18 (24.8 ± 5.21)	12	Biodentine: 89.8%
Alnassar, 2022	FP	Immature	Irreversible pulpitis	MTA (15), Bioceramic putty (15)	10	6–8 (7.34)	12	MTA: 100%; Bioceramic putty: 93.3%
Sharaan, 2022	FP	Mixed	Irreversible pulpitis	MTA (20), CEM (20)	12	7–14 (10.72 ± 2.02)	12	MTA: 100%; CEM: 100%
Tzanetakis, 2023	PP	Mature	Irreversible pulpitis	MTA (74), TotalFill BC (63)	NA	> 10 (median: 36, IQR: 21)	24	MTA: 89.2%; TotalFill BC: 82.5%
Singh, 2023	PP	Mature	Reversible pulpitis	MTA (25), Biodentine (24), CH (25), Emdogain (25)	1–2	15–45 (28.3)	12	MTA: 91.7%; CH: 91.3%; Biodentine: 100%; Emdogain: 100%
Singla, 2023	FP	Mature	Irreversible pulpitis	MTA (25), Biodentine (25)	10	18–60 (31 ± 12.6)	18	MTA: 63.6%; Biodentine: 69.6%

CEM: calcium-enriched mixture; CH: calcium hydroxide; FP: full pulpotomy; IQR: interquartile range; MTA: mineral trioxide aggregate; NA: not available; PP: partial pulpotomy; PRF: platelet-rich fibrin; SD: standard deviation.

### Risk of bias assessment

**Fig 2:**
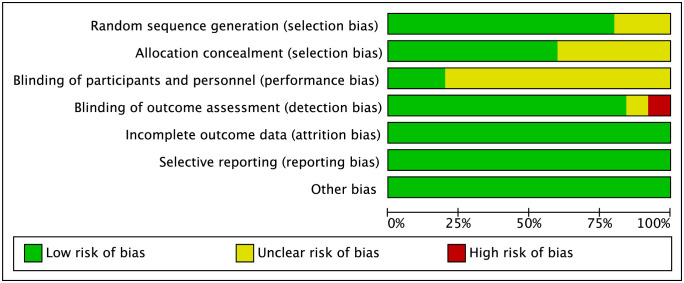
Risk of bias graph of included randomized controlled trials.

**Fig 3:**
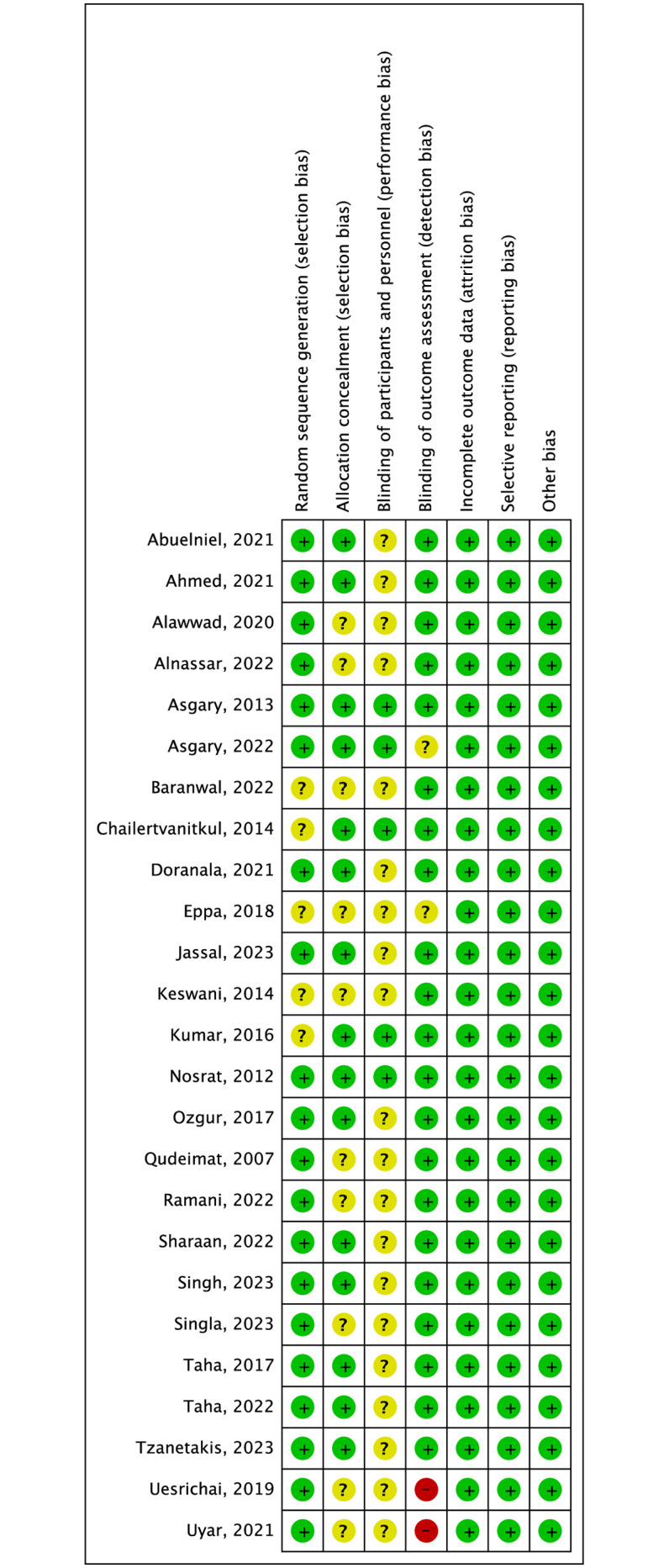
Risk of bias summary of included randomized controlled trials.

Most of the trials applied appropriate methods to generate random sequence and 15 of 25 trials described allocation concealment. Thus, 13 randomized trials were considered to have low risk of selection bias. Due to the nature of capping materials, the operators were not blinded to group assignment in most of studies with only patients blinded. These studies were considered to have unclear risk of performance bias, while the other 5 trials blinding both of operators and patients had low risk of performance bias [29, 30, 33, 34, 43]. In 2 trials, the assessors for clinical success and radiographic success were unblinded to the materials used, and a high risk of detection bias was considered [38, 50]. Finally, only 2 trials were deemed to have low risk of bias in overall as they had low risk of bias in all domains [29, 34]. The results of risk of bias assessment were graphically shown in Figs 2 and 3.

### Overall success rate of pulpotomy

**Fig 4:**
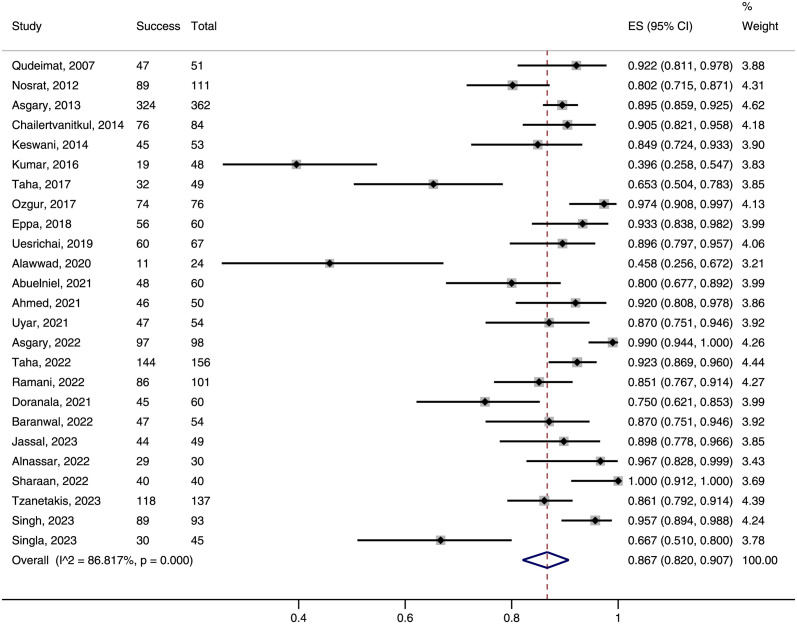
Forest plot of success rate of pulpotomy in permanent teeth with carious pulp exposure.

Among the 25 included trials, the overall success rate ranged from 39.6% to 100%, with 1743 out of 2012 evaluable teeth achieving both of clinical and radiographic success. Meta-analysis showed a pooled success rate of 86.7% (95%CI: 82.0–90.7%, Fig 4) for pulpotomy in permanent teeth with carious pulp exposure.

**Fig 5:**
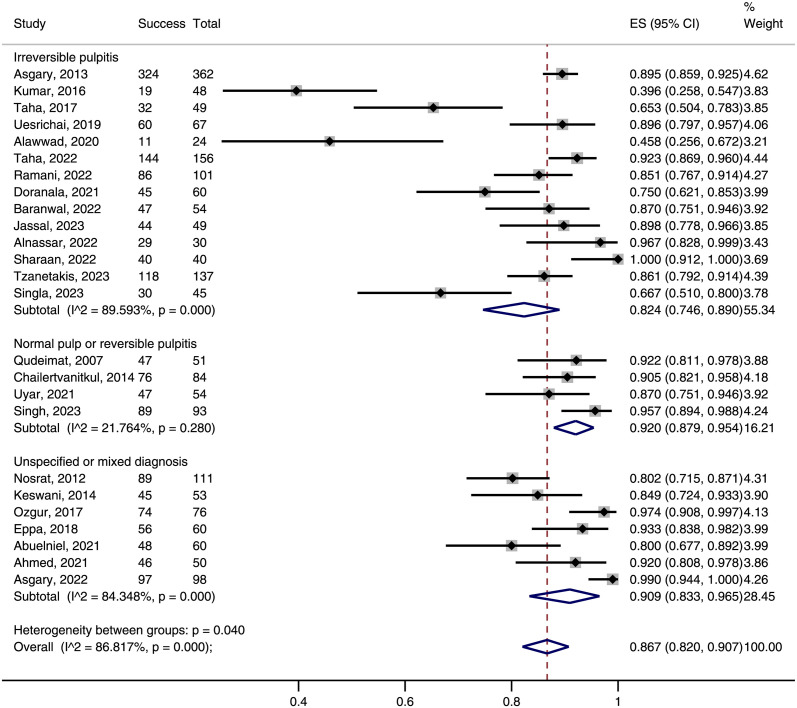
Subgroup analysis of success rate of pulpotomy stratified by preoperative pulpal diagnosis.

In mature permanent teeth, the overall success rate of pulpotomy was 83.8% (95%CI: 75.9–90.4%), which did not significantly differ from that in immature teeth (87.2% [95%CI: 80.3–92.9%], P = 0.515; S1 Fig). Similarly, the overall success rates were comparable between FP and PP (85.9% *vs* 88.2%, P = 0.566; S2 Fig) and between 12-months and 24-month assessments (85.6% *vs* 90.2%, P = 0.349; S3 Fig). When stratified by hemostasis time, the subgroup of 6 min or less cutoff had the highest success rate, followed by the subgroups of 10 min or more cutoff and unknown cutoff (S4 Fig). Yet, the difference was not statistically significant (90.4% *vs* 86.2% *vs* 80.2%, P = 0.203). We found an overall success rate of only 82.4% (95%CI: 74.6–89.0%) in subgroup of irreversible pulpitis, which was significantly lower than the 92.0% (95%CI: 87.9–95.4%) success rate in subgroup of normal pulp or reversible pulpitis (P = 0.013, Fig 5) and marginally lower than the 90.9% (95%CI: 83.3–96.5%) success rate in subgroup of unspecified or mixed diagnosis (P = 0.089, Fig 5).

Further subgroup analyses stratified by the combinations of root maturation, pulpal diagnosis, and type of pulpotomy were conducted (S2 Table). All combinations with irreversible pulpitis showed lower success rates than those with normal pulp or reversible pulpitis and those with unspecified or mixed diagnosis. Among mature teeth with irreversible pulpitis, FP and PP both achieved a similar success rate of near 80%.

### Direct comparison of capping materials

**Fig 6:**
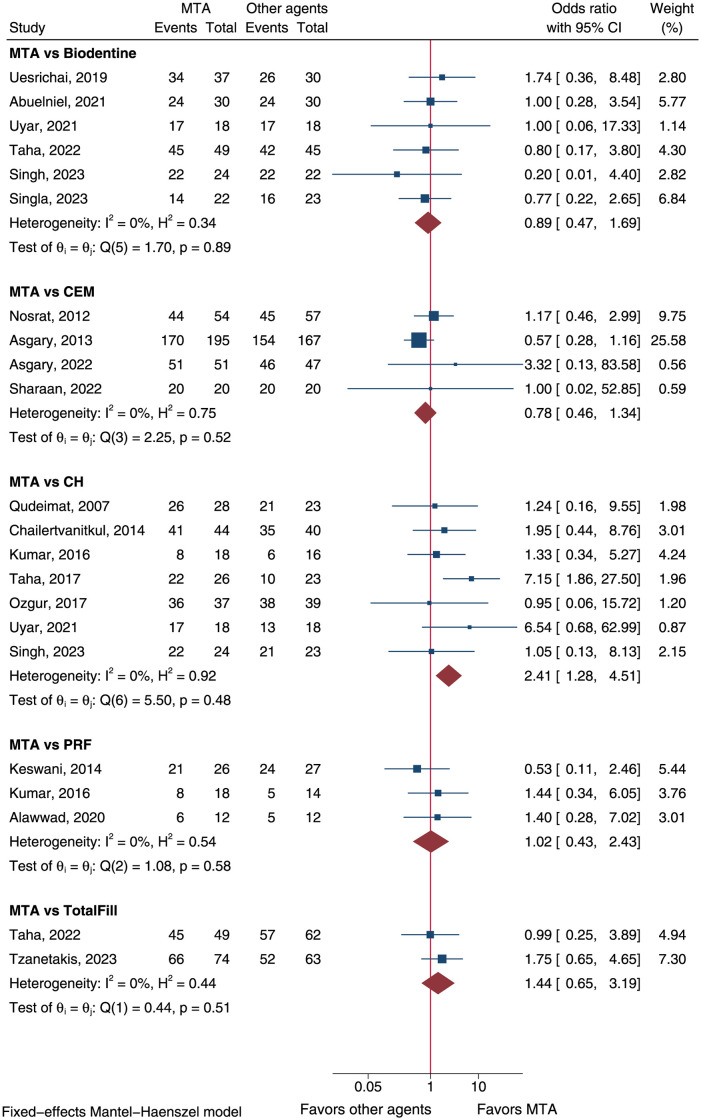
Forest plot of meta-analyses comparing MTA to other capping materials. CEM: calcium-enriched mixture; CH: calcium hydroxide; MTA: mineral trioxide aggregate; PRF: platelet-rich fibrin.

The efficacy of mineral trioxide aggregate (MTA) *vs* calcium hydroxide (CH) was compared in 7 trials [23, 30, 33, 35–37, 50]. Overall success was achieved by 172 (88.2%) of 195 subjects in MTA group and 144 (79.1%) of 182 subjects in CH group. Meta-analysis using a fixed-effect model demonstrated a significantly higher success rate in MTA than CH (OR = 2.41, 95%CI: 1.28–4.51, P = 0.006, Fig 6). Subgroup analyses showed significantly higher success rate of MTA than CH in mature teeth (OR = 2.63, 95%CI: 1.14–6.03, P = 0.023), teeth treated with partial pulpotomy (OR = 2.81, 95%CI: 1.38–5.74, P = 0.004), and in teeth diagnosed with irreversible pulpitis (OR = 3.17, 95%CI: 1.25–7.99, P = 0.014; S3 Table).

Six randomized trials compared the efficacy of MTA and Biodentine materials [23, 38, 40, 48–50]. The overall success rates of both groups were 86.7% (156/180) and 87.5% (147/168), respectively. Yet, not significant difference of success rate was found (OR = 0.89, 95%CI: 0.47–1.69, P = 0.721, Fig 6). Moreover, MTA had comparable success rate other materials, such as calcium-enriched mixture (CEM, OR = 0.78, 95%CI: 0.46–1.34, P = 0.373), platelet-rich fibrin (PRF, OR = 1.02, 95%CI: 0.43–2.43, P = 0.961), and TotalFill (OR = 1.44, 95%CI: 0.65–3.19, P = 0.370, Fig 6).

The comparison of Biodentine *vs* CH was performed in 2 trials [23, 50]. Meta-analysis suggested a higher success rate in Biodentine group than CH group at a marginal significance (OR = 6.03, 95%CI: 0.97–37.6, P = 0.054; S5 Fig). Another meta-analysis including two trials in mature teeth diagnosis with irreversible pulpitis and treated with full pulpotomy showed no difference of success rates comparing PRF *vs* CH (S6 Fig) [33, 44].

### Direct comparison of full vs partial pulpotomy

The success of full pulpotomy and partial pulpotomy was directly compared by 3 randomized trials, all of which were in mature permanent teeth with SIP [22, 45, 46]. Meta-analysis showed a trend of higher success of full pulpotomy than partial pulpotomy near the statistical significance threshold (OR = 2.16, 95%CI: 0.92–5.07, P = 0.077; S7 Fig).

### Meta-regression analysis and publication bias

Meta-regression analysis demonstrated no significant association of mean age and sample size with overall success rate (P = 0.458 and 0.299, respectively). For meta-analyses comparing MTA and CH and comparing MTA and Biodentine which had more than 5 included trials, the funnel plots were both symmetric and Egger’s test suggested no evident publication bias (P = 0.471 and 0.597, respectively).

### Certainty of evidence

The primary analysis for overall success rate was graded as very low certainty of evidence, while the comparisons of MTA *vs* Biodentine and MTA *vs* CH were both graded as low certainty of evidence (Table 2).

**Table 2:** Summary of the certainty of evidence using Grading of Recommendation Assessment, Development and Evaluation approach.

No. of studies	Certainty assessment						Effect			Certainty
	Study design	Risk of bias	Inconsistency	Indirectness	Imprecision	Publication bias	No. of events	No. of individuals	Effect size (95%CI)	
Overall success rate of pulpotomy										
25	RCT	Serious ^a^	Very serious ^b^	Serious ^c^	Not serious	None	1743	2012	Success rate = 0.867 (0.820–0.907)	⊕◯◯◯
										Very low
Comparison of different capping materials (MTA *vs* Biodentine)										
6	RCT	Serious ^a^	Not serious	Not serious	Serious ^d^	None	MTA: 156	MTA: 180	OR = 0.89 (0.47-	⊕⊕◯◯
							Biodentine: 147	Biodentine: 168	1.68)	Low
Comparison of different capping materials (MTA *vs* CH)										
7	RCT	Serious ^a^	Not serious	Not serious	Serious ^d^	None	MTA: 172	MTA: 195	OR = 2.41 (1.28–4.51)	⊕⊕◯◯
							CH: 144	CH: 182		Low

^a^ Most of included trials had moderate to high risk of bias.

^b^ Substantial between-study heterogeneity was observed.

^c^ The trials were not designed to assess the overall success rate of pulpotomy.

^d^ The total number of subjects was less than 400.

## Discussion

The present meta-analysis aimed to assess the success rate of pulpotomy in cariously exposed permanent teeth and explore the influential factors. A total of 25 eligible randomized controlled trials were included, most of which had unclear or high risk of bias. The meta-analysis demonstrated a high overall success rate (86.7%) of pulpotomy at a very low certainty of evidence. Concerning the dressing materials, MTA and Biodentine had a higher success rate than the traditional calcium hydroxide at a low certainty of evidence. Direct comparison showed a trend towards higher success rate in FP than PP. Our study suggested that pulpotomy can be effectively used as an alternative to RCT for the treatment of cariously exposed permanent teeth.

Despite a high overall success rate, the identification of prognostic factors potentially influencing pulpotomy outcomes is important to clinical decision-making [3]. Generally, immature teeth with open apices have more profuse blood supply and better regenerative potential than mature teeth with closed apices [51]. Pulpotomy is expected to have more favorable outcomes in immature teeth, but there is limited supporting clinical evidence. Our meta-analysis revealed a 83.8% success in mature teeth and 87.2% success in immature teeth without significant difference. Event controlling for pulpotomy type and pulpal diagnosis, the success rates did not significantly differ between both groups. Therefore, pulpotomy is effective for permanent teeth with either open apices or closed apices.

Irreversible pulpitis, including symptomatic (SIP) and asymptomatic (AIP), indicates incapability of healing of vital pulp and is usually recommended for RCT treatment. Previous meta-analysis of randomized clinical trials have demonstrated promising outcomes and high success rates of pulpotomy, indicating that pulpotomy is suitable for definitive treatment of mature teeth with irreversible pulpitis [18, 20]. Our analysis yielded a 82.4% success rate in teeth diagnosed with irreversible pulpitis and a 79.8% success rate in mature teeth with irreversible pulpitis, respectively. Yet, the success rates in teeth with irreversible pulpitis were significantly lower than the rates in teeth with normal pulp or reversible pulpitis. These results were consistent with findings from another meta-analysis [17]. Elmsmari *et al*. performed a meta-analysis that included 5 randomized trials and 6 prospective studies [17]. They revealed a 75% success rate of partial pulpotomy to treat cariously exposed permanent teeth diagnosed with irreversible pulpitis at 1 year, which was significantly lower than that in teeth with reversible pulpitis [17]. Nevertheless, the success rate of pulpotomy in irreversible pulpitis is exceeding 80%, suggesting that pulpotomy is also effective in treating cariously exposed teeth with irreversible pulpitis. Nowadays, pulpotomy has been recommended as an alternative option to conventional RCT in the management of definitive mature teeth with SIP [1].

It should be noted that the pulp diagnosis is highly inaccurate and subjective, challenging the use of pre-operative pulp diagnosis as an indicator of VPT. The pulp condition, usually pre-operatively judged according to patient’s pain history and appropriate clinical tests, should be intra-operatively confirmed by the assessment of pulpal bleeding, tissue color and consistency [52]. Taha *et al*. found 7 pulps with symptoms indicative of irreversible pulpitis were necrotic as demonstrated by the absence of bleeding tissue after pulp exposure [53]. In Nosrat’s study, 4 teeth with pre-operative diagnosis of irreversible pulpitis showed absence of bleeding upon exposure [34]. The necrotic teeth determined by intro-operative assessment were then excluded from VPT procedure. These results suggest that pre-operative assessment alone on pulp status may overestimate pulp’s amenability to VPT.

Another important factor influencing pulpotomy outcomes is the pulp capping materials [6]. For many years, calcium hydroxide (CH) has been considered as the first choice of capping materials in pulp capping owing to its antimicrobial nature and ability to promote hard tissue formation [54, 55]. However, the application of CH encounters some barriers concerning the its toxicity, high solubility, presence of tunnel defect in dentine bridges, and low mechanical resistance [56, 57]. Several studies have revealed the success rate of CH in treating cariously exposed permanent teeth is lower than 80% and declines over time [58, 59]. In recent years, hydrophilic calcium silicate-based cement, such as MTA, Biodentine and CEM, has become a promising capping material for pulpotomy with encouraging results [30, 50, 60]. MTA has several advantages over CH as a capping material, such as biocompatibility, low solubility, superior sealing property, antimicrobial ability, and lower chance of tunnel defect [61]. Biodentine is developed to overcome the drawbacks of MTA like tooth discoloration and prolonged setting time [62, 63]. CEM is another bioceramic material with similar property but shorter setting time than MTA [64]. Direct comparisons in meta-analysis demonstrated a significantly higher success rate of MTA than CH (P = 0.006) and a higher success rate of Biodentine than CH at marginal significance (P = 0.054). No significant differences of success rates were found when comparing MTA to Biodentine or CEM. Therefore, biomaterials such as MTA, Biodentine and CEM can be recommended as pulpotomy capping materials in permanent teeth with carious pulp exposure.

For cariouly exposed permanent teeth, FP is to completely remove the coronal pulp with a biocompatible capping material placed onto the pulping tissue at the level of the canal orifice [6]. PP is to remove a small proportion of pulp tissue (usually to a depth of approximately 2–3 mm of exposed pulp) with a dressing material onto the remaining pulp tissue [6]. Compared with PP, FP has a higher chance of complete removal of infected and inflammatory pulp tissue [53]. Yet, FP may inhibit dentinal development and result in root canal obliteration, especially in immature teeth [65]. Calcification of canal space is not rare and unresponsiveness to sensibility test is frequently seen in FP [66]. Our meta-analysis demonstrated similar success rates between FP and PP. However, this result may be confounded by the preoperative pulp status and is not a direct comparison. Recently, three randomized trials directly compared both pulpotomy procedures in cariously exposed pulps of mature teeth with SIP [22, 45, 46]. Two trials, performed by Ramani *et al*. and Baranwal *et al*. respectively, both revealed a slightly but non-significantly higher success rate of FP than PP at 1 year (89.8% *vs* 80.8%; 92.8% *vs* 80.7%) [22, 46]. Meta-analysis pooling these 3 trials suggested a non-significant trend of higher success rate of full pulpotomy than partial pulpotomy (OR = 2.16, P = 0.077). This result should be cautiously interpreted as the total sample size is small. Overall, FP and PP have yielded similar success rate in cariously exposed permanent teeth, and more well-designed and large-scale trials are warranted, especially in the scenario of SIP diagnosis, in the future.

Our study has several limitations. There is substantial between-study heterogeneity. This may be caused by the great variations in clinical features in terms of age, root apices development and pulpal diagnosis, pulpotomy procedures regarding pulpotomy type, depth of pulp removal, hemostasis and final restoration materials, and the outcome assessment criteria. Despite the inclusion of many eligible randomized trials, the analysis of each direct comparison of two capping materials were conducted that only included several trials with a small sample size. Moreover, most of the included trials had moderate to high risk of bias. Owing to the above reasons, the conclusions of our results only had very low to low certainty of evidence.

## Conclusions

The present meta-analysis suggested that pulpotomy is an effective definitive treatment for permanent teeth with carious pulp exposure regardless of pre-operative pulp inflammation. MTA and Biodentine, with superior performance than the conventional CH, can be recommended as capping materials.

## Supporting information

S1 Checklist PRISMA 2020 checklist. (DOCX)

S1 Table List of excluded studies with reasons. (DOCX)

S2 Table Subgroup analysis of success rate stratified by the combination of root maturation, pulpal diagnosis, and type of pulpotomy. (DOCX)

S3 Table Subgroup analysis for the comparison between MTA and CH. (DOCX)

S1 Fig Subgroup analysis of success rate of pulpotomy stratified by root maturation. (PDF)

S2 Fig Subgroup analysis of success rate of pulpotomy stratified by type of pulpotomy. (PDF)

S3 Fig Subgroup analysis of success rate of pulpotomy stratified by duration of follow-up. (PDF)

S4 Fig Subgroup analysis of success rate of pulpotomy stratified by hemostasis time. (PDF)

S5 Fig Forest plot of meta-analysis comparing Biodentine to CH. CH: calcium hydroxide.
(PDF)

S6 Fig Forest plot of meta-analysis comparing PRF to CH. PRF: platelet-rich fibrin; CH: calcium hydroxide.
(PDF)

S7 Fig Forest plot of meta-analysis comparing FP to PP. FP: full pulpotomy; PP: partial pulpotomy.
(PDF)
