# Supplementary material for: Efficacy of pulpotomy for permanent teeth with carious pulp exposure: A systematic review and meta-analysis of randomized controlled trials
Source: PLoS One. 2024 Jul 5;19(7):e0305218. doi: 10.1371/journal.pone.0305218 (PMC11226044; doi:10.1371/journal.pone.0305218)
Supplement: S1 Table — (DOCX) [file pone.0305218.s002.docx]

S1 Table. List of excluded studies with reasons.

| Study | Title | Reason for exclusion |
| --- | --- | --- |
| Abuelniel, 2020 | A comparison of MTA and Biodentine as medicaments for pulpotomy in traumatized anterior immature permanent teeth: A randomized clinical trial | Including traumatized permanent teeth |
| Al-Batayneh, 2022 | Outcome of vital pulp therapy in deeply carious molars affected with molar incisor hypomineralisation (MIH) defects: a randomized clinical trial | Comparing pulpotomy to indirect pulp capping |
| Asgary, 2013 | One-year results of vital pulp therapy in permanent molars with irreversible pulpitis: an ongoing multicenter, randomized, non-inferiority clinical trial | Comparing pulpotomy to root canal therapy |
| Asgary, 2014 | Two-year results of vital pulp therapy in permanent molars with irreversible pulpitis: an ongoing multicenter randomized clinical trial | Comparing pulpotomy to root canal therapy |
| Asgary, 2015 | Five-year results of vital pulp therapy in permanent molars with irreversible pulpitis: a non-inferiority multicenter randomized clinical trial | Comparing pulpotomy to root canal therapy |
| Cengiz, 2016 | Efficacy of Erbium, Chromium-doped:Yttrium, Scandium, Gallium, and Garnet Laser Irradiation Combined with Resin-based Tricalcium Silicate and Calcium Hydroxide on Direct Pulp Capping: A Randomized Clinical Trial | Comparing different capping materials in direct pulp capping |
| Eghbal, 2020 | Postendodontic Pain after Pulpotomy or Root Canal Treatment in Mature Teeth with Carious Pulp Exposure: A Multicenter Randomized Controlled Trial | Comparing pulpotomy to root canal therapy |
| Eid, 2022 | Effectiveness of Three Agents in Pulpotomy Treatment of Permanent Molars with Incomplete Root Development: A Randomized Controlled Trial | Including traumatized permanent teeth |
| Elchaghaby, 2020 | Clinical and radiographic evaluation of indirect pulp treatment of young permanent molars using photo-activated oral disinfection versus calcium hydroxide: a randomized controlled pilot trial | Comparing different capping materials in indirect pulp capping |
| El-Meligy, 2006 | Comparison of mineral trioxide aggregate and calcium hydroxide as pulpotomy agents in young permanent teeth (apexogenesis) | Including traumatized permanent teeth |
| Esteve-Pardo, 2023 | Pulpectomy vs. Pulpotomy as Alternative Emergency Treatments for Symptomatic Irreversible Pulpitis-A Multicenter Comparative Randomised Clinical Trial on Patient Perceptions | Comparing pulpotomy to pulpectomy |
| Galani, 2017 | Comparative Evaluation of Postoperative Pain and Success Rate after Pulpotomy and Root Canal Treatment in Cariously Exposed Mature Permanent Molars: A Randomized Controlled Trial | Comparing pulpotomy to root canal therapy |
| Ghoddusi, 2012 | Clinical and radiographic evaluation of vital pulp therapy in open apex teeth with MTA and ZOE | Including traumatized permanent teeth |
| Guang, 2022 | Comparison of pulpal vitalization and root canal therapy in symptomatic immature permanent molars | Only reported radiographical outcomes |
| Hegde, 2017 | Clinical evaluation of mineral trioxide aggregate and biodentine as direct pulp capping agents in carious teeth | Comparing different capping materials in direct pulp capping |
| Hilton, 2013 | Comparison of CaOH with MTA for direct pulp capping: a PBRN randomized clinical trial | Comparing different capping materials in direct pulp capping |
| Iyer, 2021 | Comparative evaluation of different direct pulp capping agents in carious tooth: An in vivo study | Comparing different capping materials in direct pulp capping |
| Kang, 2017 | A randomized controlled trial of various MTA materials for partial pulpotomy in permanent teeth | Including traumatized permanent teeth |
| Sawicki, 2008 | Histological evaluation of mineral trioxide aggregate and calcium hydroxide in direct pulp capping of human immature permanent teeth | Comparing different capping materials in direct pulp capping |
| Selvendran, 2022 | Comparison of three different materials used for indirect pulp capping in permanent molars: An in vivo study | Comparing different capping materials in indirect pulp capping |
| Suhag, 2019 | Success of Direct Pulp Capping Using Mineral Trioxide Aggregate and Calcium Hydroxide in Mature Permanent Molars with Pulps Exposed during Carious Tissue Removal: 1-year Follow-up | Comparing different capping materials in direct pulp capping |
| Taha, 2023 | A Randomized Controlled Clinical Trial of Pulpotomy versus Root Canal Therapy in Mature Teeth with Irreversible Pulpitis: Outcome, Quality of Life, and Patients' Satisfaction | Comparing pulpotomy to root canal therapy |
| Tozar, 2020 | Evaluation of the Efficacy of Erbium, Chromium-doped Yttrium, Scandium, Gallium, and Garnet Laser in Partial Pulpotomy in Permanent Immature Molars: A Randomized Controlled Trial | Comparing MTA to MTA + Laser |
| Tquiq, 2019 | Pulpotomy of Immature Permanent Teeth using Two Different Sealing Materials: A Comparative Study | Including traumatized permanent teeth |
| Vu, 2020 | Acemannan Used as an Implantable Biomaterial for Vital Pulp Therapy of Immature Permanent Teeth Induced Continued Root Formation | Including traumatized permanent teeth |
| Yang, 2020 | The effect of partial pulpotomy with iRoot BP Plus in traumatized immature permanent teeth: A randomized prospective controlled trial | Including traumatized permanent teeth |
| Yazdanfar, 2020 | Combination effects of diode laser and resin-modified tricalcium silicate on direct pulp capping treatment of caries exposures in permanent teeth: a randomized clinical trial | Comparing different capping materials in direct pulp capping |
