# Supplementary material for: Efficacy of pulpotomy for permanent teeth with carious pulp exposure: A systematic review and meta-analysis of randomized controlled trials
Source: PLoS One. 2024 Jul 5;19(7):e0305218. doi: 10.1371/journal.pone.0305218 (PMC11226044; doi:10.1371/journal.pone.0305218)
Supplement: S2 Table — (DOCX) [file pone.0305218.s003.docx]

S2 Table. Subgroup analysis of success rate stratified by the combination of root maturation, pulpal diagnosis, and type of pulpotomy.

| Combination | No. of trials | Success rate (%) | 95%CI (%) | I^2^ (%) |
| --- | --- | --- | --- | --- |
| Pulpotomy type × root maturation |  |  |  |  |
| FP, mature teeth | 9 | 84.6 | 73.9-93.0 | 92.1 |
| FP, immature teeth | 7 | 84.4 | 74.8-92.1 | 80.3 |
| PP, mature teeth | 6 | 84.2 | 74.5-92.1 | 79.3 |
| PP, immature teeth | 3 | 92.4 | 85.1-97.6 | 64.9 |
| Pulpotomy type × pulpal diagnosis |  |  |  |  |
| FP, irreversible pulpitis | 12 | 83.7 | 74.1-91.6 | 90.3 |
| FP, normal pulp or reversible pulpitis | 0 | - | - | - |
| FP, unspecified or mixed diagnosis | 5 | 90.3 | 80.1-97.3 | 87.0 |
| PP, irreversible pulpitis | 6 | 82.6 | 75.4-88.9 | 58.9 |
| PP, normal pulp or reversible pulpitis | 4 | 92.0 | 87.9-95.4 | 21.8 |
| PP, unspecified or mixed diagnosis | 1 | - | - | - |
| Root maturation × pulpal diagnosis |  |  |  |  |
| Mature teeth, irreversible pulpitis | 10 | 79.8 | 71.2-87.3 | 89.5 |
| Mature teeth, normal pulp or reversible pulpitis | 1 | - | - | - |
| Mature teeth, unspecified or mixed diagnosis | 1 | - | - | - |
| Immature teeth, irreversible pulpitis | 2 | 79.0 | 66.7-89.2 | 0 |
| Immature teeth, normal pulp or reversible pulpitis | 2 | 89.2 | 83.4-94.0 | 0 |
| Immature teeth, unspecified or mixed diagnosis | 6 | 88.8 | 81.6-94.5 | 75.8 |
| Mature teeth, FP, irreversible pulpitis | 8 | 81.7 | 70.2-90.9 | 90.8 |
| Mature teeth, PP, irreversible pulpitis | 5 | 80.8 | 72.3-88.1 | 58.2 |

FP: full pulpotomy; PP: partial pulpotomy; SIP: symptomatic irreversible pulpitis.
