# Supplementary material for: Efficacy of pulpotomy for permanent teeth with carious pulp exposure: A systematic review and meta-analysis of randomized controlled trials
Source: PLoS One. 2024 Jul 5;19(7):e0305218. doi: 10.1371/journal.pone.0305218 (PMC11226044; doi:10.1371/journal.pone.0305218)
Supplement: S3 Table — (DOCX) [file pone.0305218.s004.docx]

S3 Table. Subgroup analysis for the comparison between MTA and CH.

| Subgroup | No. of trials | OR (95%CI) | P |
| --- | --- | --- | --- |
| Root maturation |  |  |  |
| Mature | 3 | 2.63 (1.14-6.03) | 0.023 |
| Immature | 3 | 2.50 (0.83-7.51) | 0.103 |
| Mixed | 1 | - | - |
| Type of pulpotomy |  |  |  |
| FP | 1 | - | - |
| PP | 6 | 2.81 (1.38-5.74) | 0.004 |
| Pulpal diagnosis |  |  |  |
| Irreversible pulpitis | 2 | 3.17 (1.25-7.99) | 0.014 |
| Normal pulp or reversible pulpitis | 4 | 2.03 (0.82-5.05) | 0.127 |
| Unspecified or mixed diagnosis | 1 | - | - |

CH: calcium hydroxide; MTA: mineral trioxide aggregate; OR: odds ratio
