# Supplementary figures and images for: Efficacy of pulpotomy for permanent teeth with carious pulp exposure: A systematic review and meta-analysis of randomized controlled trials
Source: PLoS One. 2024 Jul 5;19(7):e0305218. doi: 10.1371/journal.pone.0305218 (PMC11226044; doi:10.1371/journal.pone.0305218)

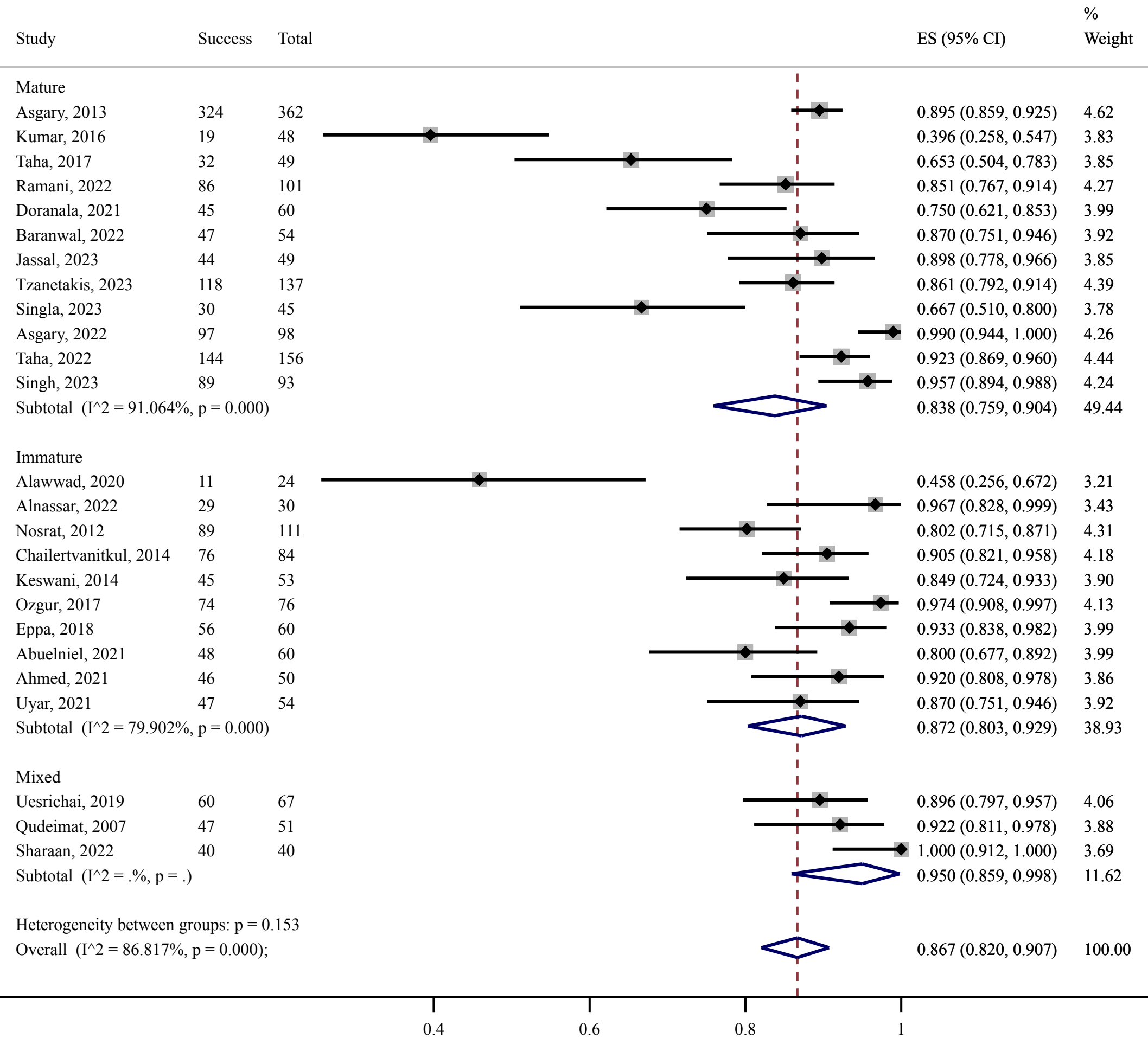

Supplement: S1 Fig — (PDF) [file pone.0305218.s005.pdf]

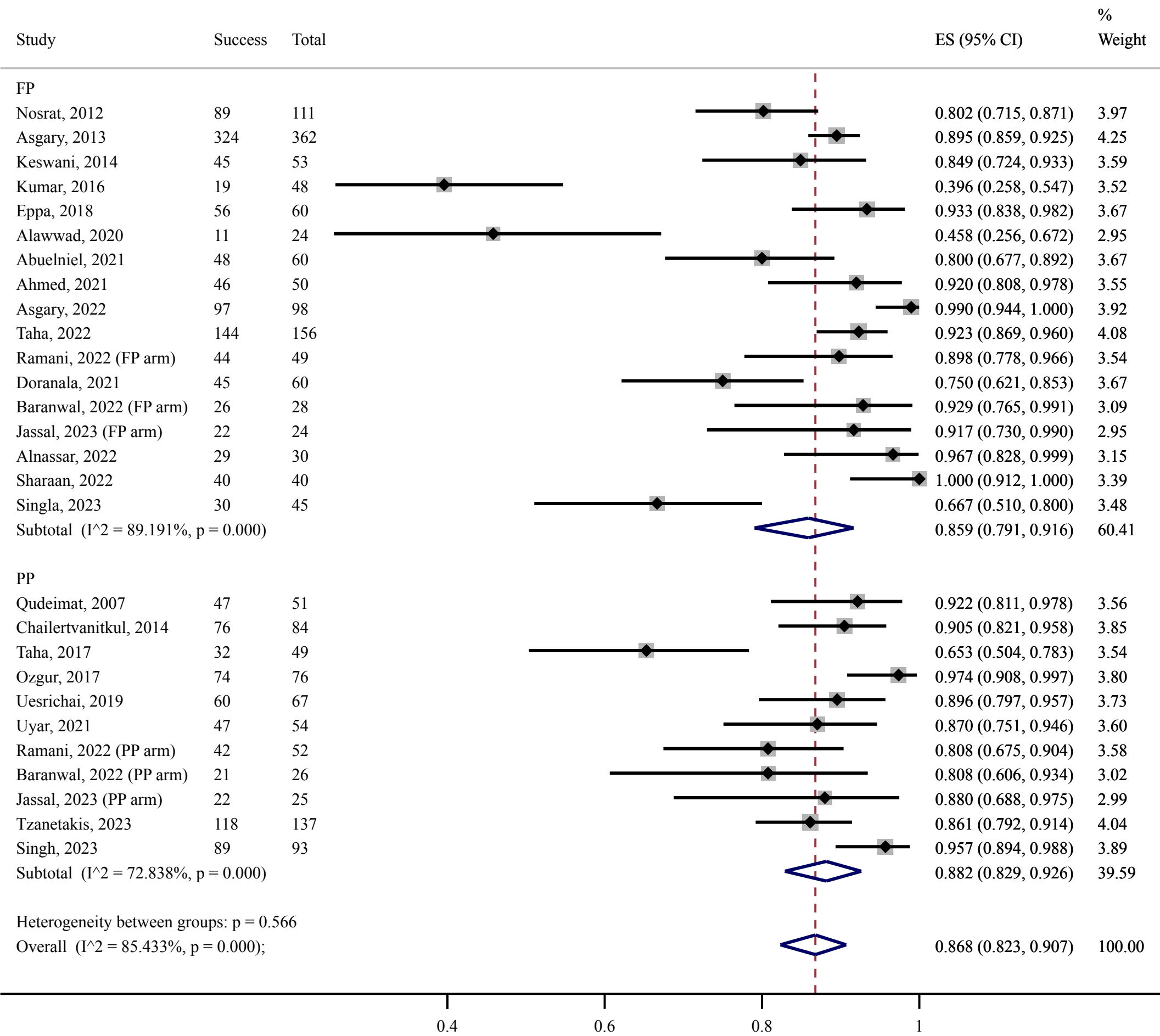

Supplement: S2 Fig — (PDF) [file pone.0305218.s006.pdf]

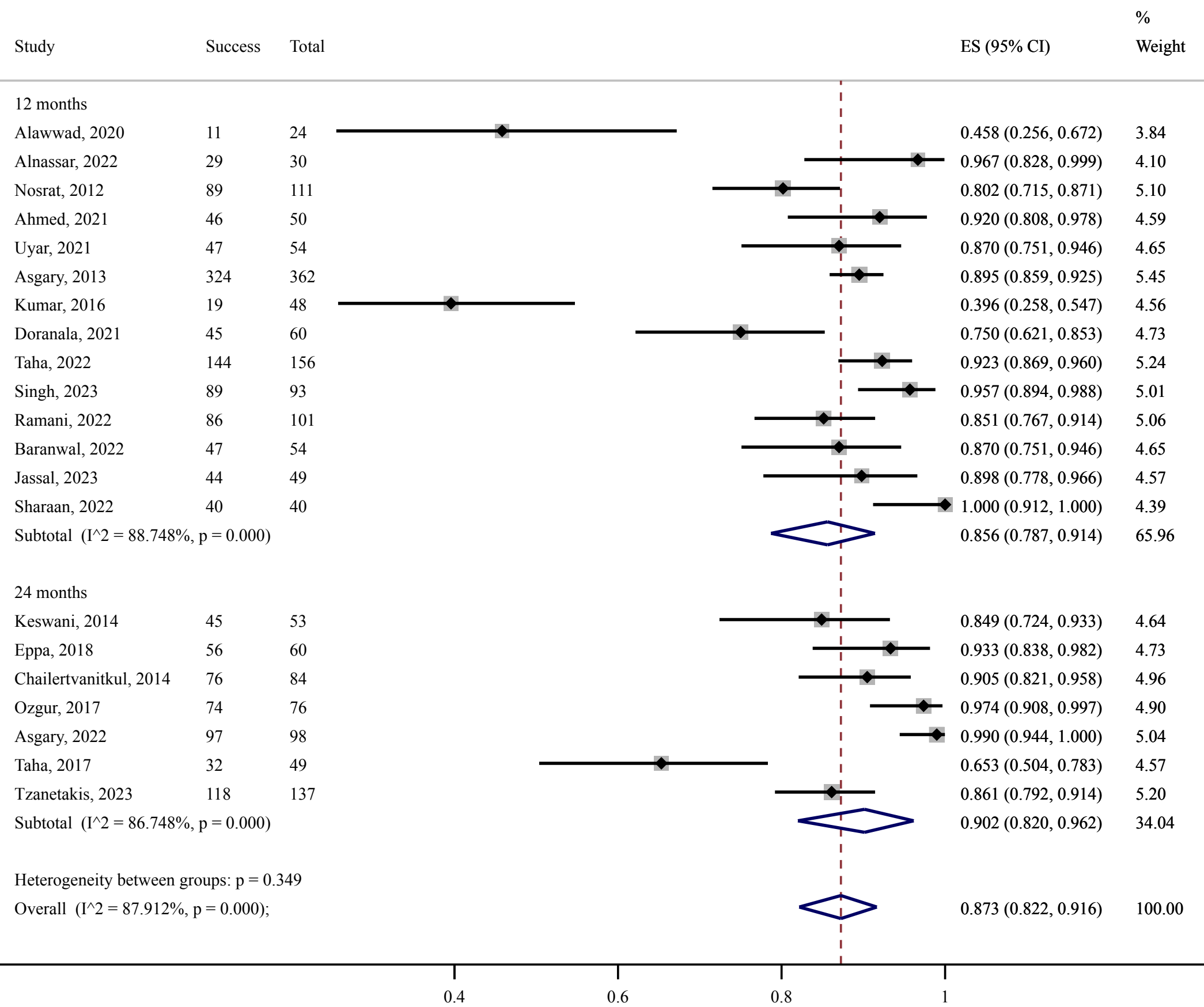

Supplement: S3 Fig — (PDF) [file pone.0305218.s007.pdf]

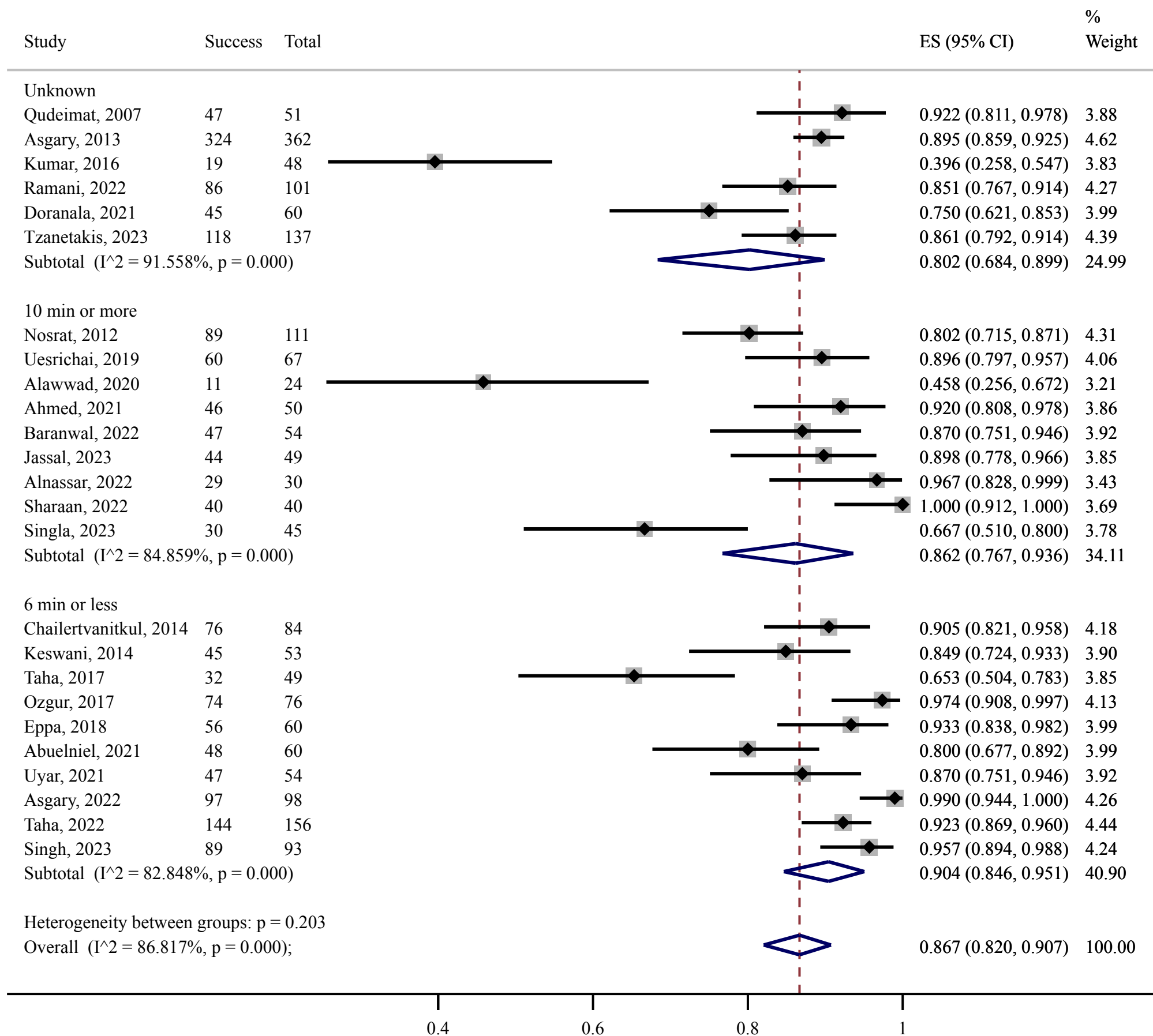

Supplement: S4 Fig — (PDF) [file pone.0305218.s008.pdf]

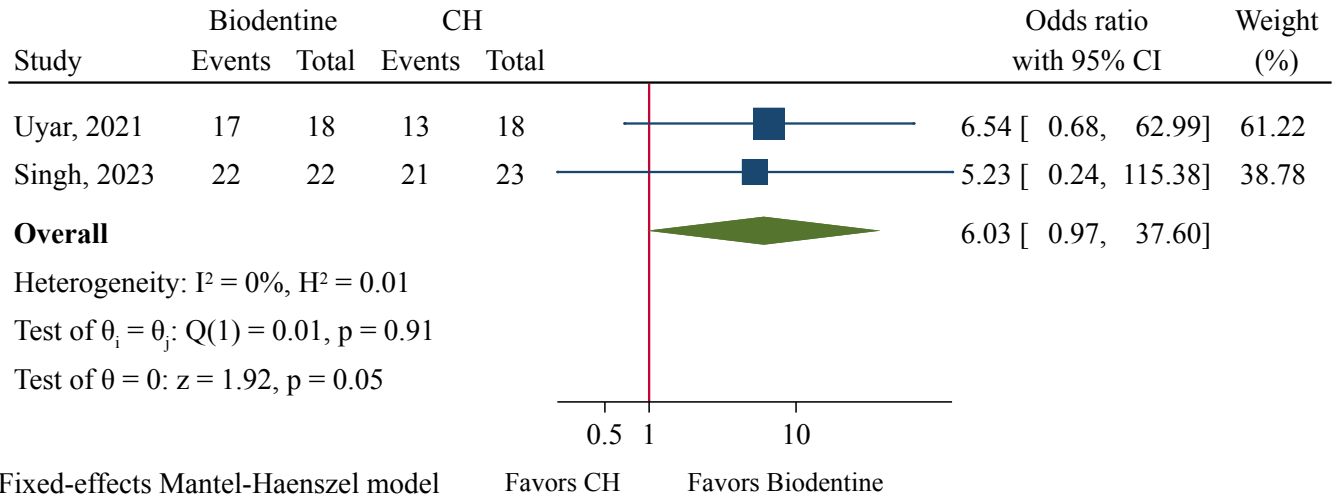

Supplement: S5 Fig — CH: calcium hydroxide. (PDF) [file pone.0305218.s009.pdf]

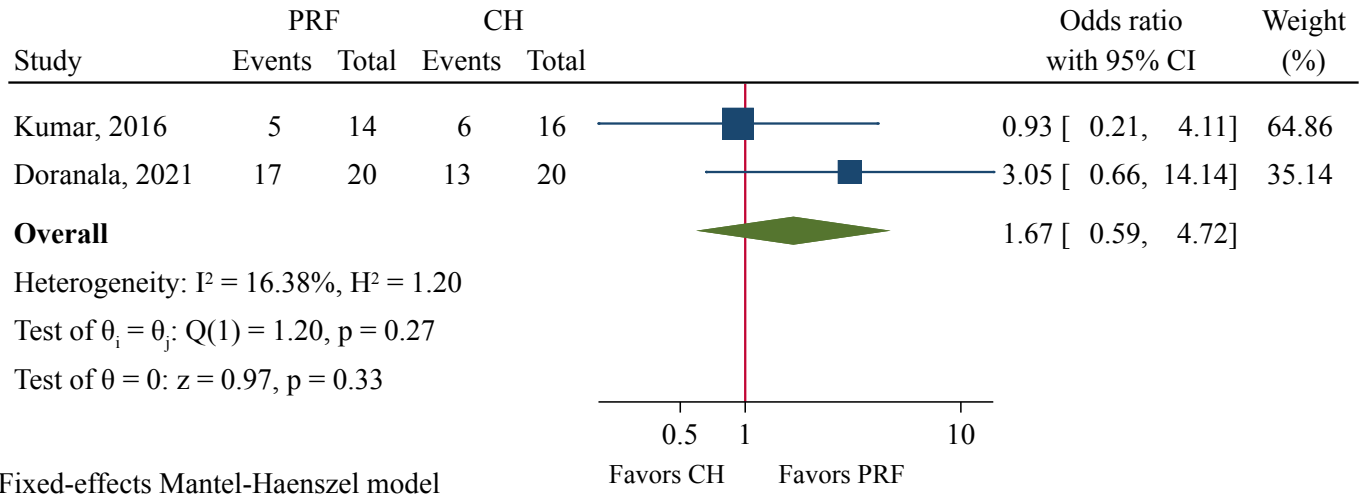

Supplement: S6 Fig — PRF: platelet-rich fibrin; CH: calcium hydroxide. (PDF) [file pone.0305218.s010.pdf]

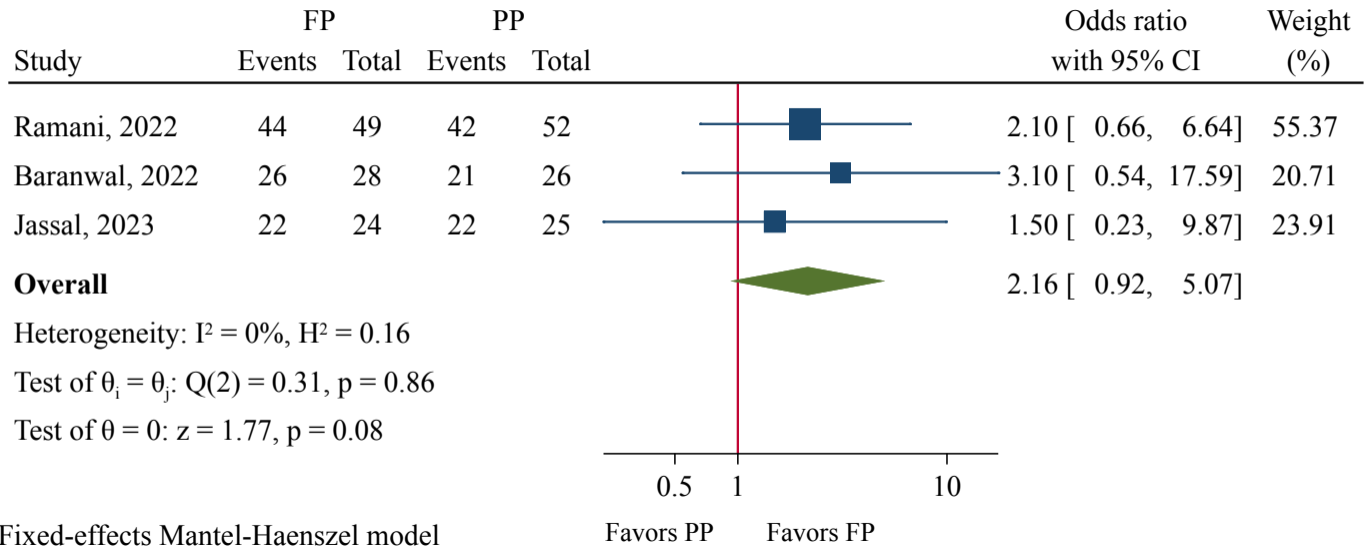

Supplement: S7 Fig — FP: full pulpotomy; PP: partial pulpotomy. (PDF) [file pone.0305218.s011.pdf]
